# Structural insights into the mechanism defining substrate affinity in *Arabidopsis thaliana* dUTPase: the role of tryptophan 93 in ligand orientation

**DOI:** 10.1186/s13104-015-1760-1

**Published:** 2015-12-15

**Authors:** Noriko Inoguchi, Kittichai Chaiseeda, Mamoru Yamanishi, Moon Ki Kim, Yunho Jang, Mamta Bajaj, Catherine P. Chia, Donald F. Becker, Hideaki Moriyama

**Affiliations:** School of Biological Sciences, University of Nebraska-Lincoln, Lincoln, NE USA; Department of Chemistry, University of Nebraska-Lincoln, Lincoln, NE USA; School of Mechanical Engineering, Sungkyunkwan University, 300 Cheoncheon, Suwon, South Korea; Department of Mechanical and Industrial Engineering, University of Massachusetts, Amherst, MA USA; Department of Biochemistry, University of Nebraska-Lincoln, Lincoln, NE USA; Virology, Surveillance and Diagnostic Branch, Influenza Division, Centers for Disease Control and Prevention, Atlanta, GA USA

**Keywords:** Deoxyuridine triphosphate nucleotidohydrolase, Substrate affinity, Drug targets, *Arabidopsis thaliana*

## Abstract

**Background:**

Deoxyuridine triphosphate nucleotidohydrolase (dUTPase) hydrolyzes dUTP to dUMP and pyrophosphate to maintain the cellular thymine-uracil ratio. dUTPase is also a target for cancer chemotherapy. However, the mechanism defining its substrate affinity remains unclear. Sequence comparisons of various dUTPases revealed that *Arabidopsis thaliana* dUTPase has a unique tryptophan at position 93, which potentially contributes to its degree of substrate affinity. To better understand the roles of tryptophan 93, *A. thaliana* dUTPase was studied.

**Results:**

Enzyme assays showed that *A. thaliana* dUTPase belongs to a high-affinity group of isozymes, which also includes the enzymes from *Escherichia coli* and *Mycobacterium tuberculosis*. Enzymes from *Homo sapiens* and *Saccharomyces cerevisiae* are grouped as low-affinity dUTPases. The structure of the homo-trimeric *A. thaliana* dUTPase showed three active sites, each with a different set of ligand interactions between the amino acids and water molecules. On an α-helix, tryptophan 93 appears to keep serine 89 in place via a water molecule and to specifically direct the ligand. Upon being oriented in the active site, the C-terminal residues close the active site to promote the reaction.

**Conclusions:**

In the high-affinity group, the prefixed direction of the serine residues was oriented by a positively charged residue located four amino acids away, while low-affinity enzymes possess small hydrophobic residues at the corresponding sites.

**Electronic supplementary material:**

The online version of this article (doi:10.1186/s13104-015-1760-1) contains supplementary material, which is available to authorized users.

## Background

Deoxyuridine triphosphate nucleotidohydrolase (dUTPase; EC 3.6.1.23) is an important enzyme that prevents uracil misincorporation during *de novo* DNA synthesis [1]. It catalyzes the hydrolysis of dUTP to deoxyuridine monophosphate (dUMP) and inorganic pyrophosphate [2, 3], thereby maintaining an appropriate level of dUTP with respect to deoxythymidine triphosphate (dTTP) levels [4]. Compromising dUTPase activity in fast-growing cells causes an imbalance in the dUTP–dTTP ratio that can cause uracil misincorporation into DNA [1]. Due to its role in fast-growth-specific cell death, dUTPase has been a target for cancer chemotherapy [5, 6].

**Fig. 1:**
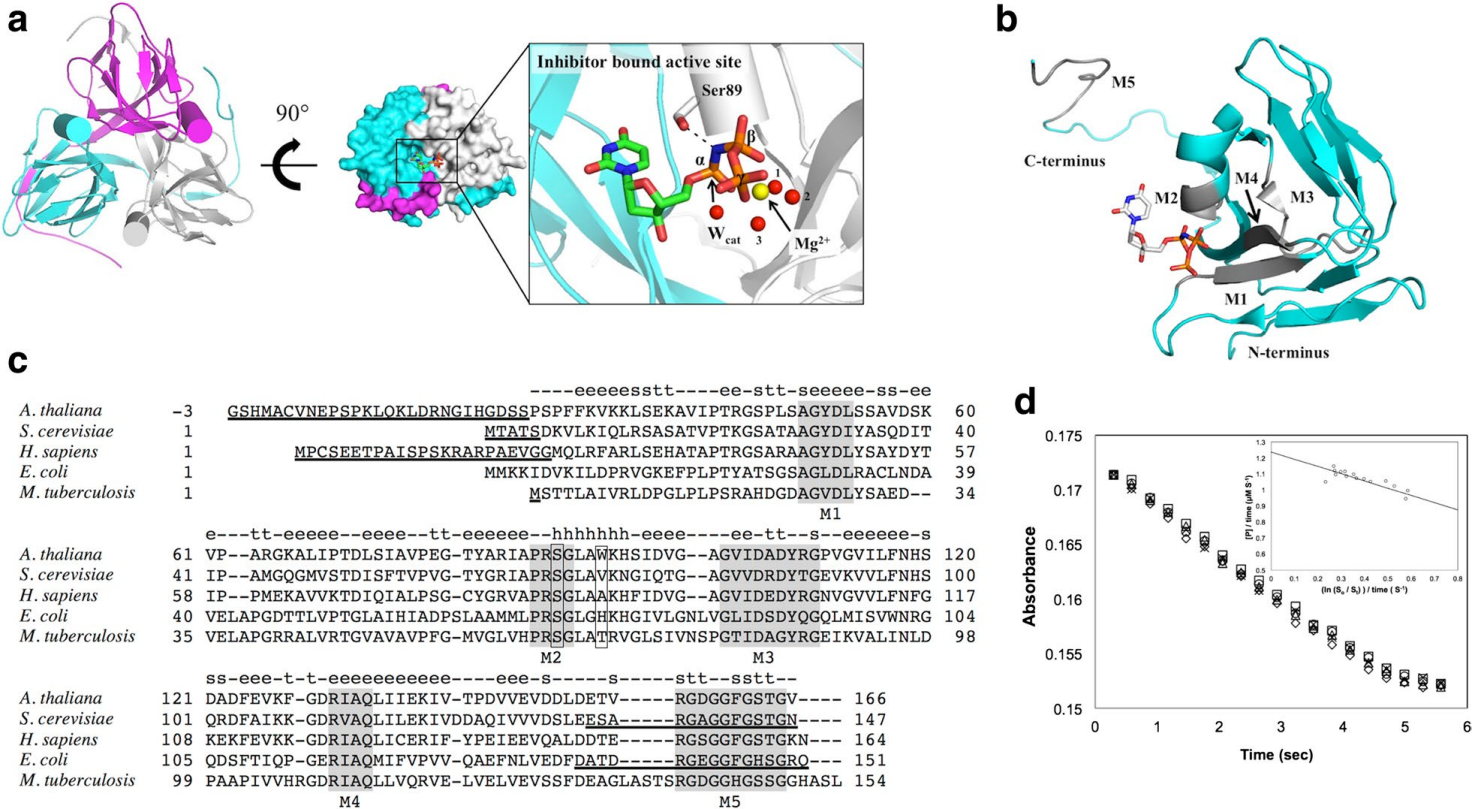
The structure of dUTPase. **a** The structure of *Arabidopsis* dUTPase with an inhibitor bound at the active site. The *cylinders* in each subunit represent only one helix, which contains Ser89 and Trp93. **b** Each subunit shows five conserved motifs. The five conserved motifs (M1–M5) are highlighted in *gray* in the holo *Arabidopsis* dUTPase chain B. **c** Sequence alignment of dUTPase. The five conserved motifs are indicated by *gray shading*. Ser89 and Trp93 are *boxed*. The secondary structure of chain B in the holo *Arabidopsis* dUTPase, which was identified using DSSP [27], is indicated by the *lowercase letters in the top line*; ‘e’, β-strand; ‘t’, hydrogen bonded turns; ‘s’, bend; and ‘h’, α-helix. The coordinates of the *underlined amino acid* residues were not modeled (Table 1). **d** Estimation of kinetic parameters. Five independent data sets were plotted with different symbols. The *inset graph* shows the integrated Michaelis–Menten equation fit to the absorbance data shown. The *solid line* in the figure shows the best-fit line used to estimate the *K*_m_ value

Homo-trimeric dUTPases have three active sites, each of which consists of five conserved motifs [3] (Fig. 1a–c). An aspartate in motif 1 interacts with active site water molecules to stabilize the divalent cation cofactor Mg^2+^, which is important for enzymatic activity [7–9]. A serine in motif 2 interacts with the oxygen atom between α, β-phosphate to induce a reaction-favorable orientation [10] (Ser 89 in *Arabidopsis* dUTPase). An aspartate in motif 3 activates catalytic water [11]; a glutamine in motif 4 also interacts with the catalytic water (Wcat in Fig. 1a) and the ligand [9]. Interactions between ligands and residues in motif 5 help orient the ligand so that the α phosphate locates close to the catalytic water [12]. The homo-trimeric dUTPase kinetic mechanism has mainly been studied by multidimensional nuclear magnetic resonance (NMR) [13, 14], quench-flow experiments [15], and the mixed quantum mechanics/molecular mechanics (QM/MM) calculations [16]. These studies have revealed at least four distinct enzymatic steps including substrate binding, isomerization, hydrolysis, and release. However, the mechanism for defining the substrate affinity remains unclear.

To address the basis for substrate affinity differences among dUTPases, we chose those five dUTPases using two criteria, including (i) consistent measurement conditions of K_m_ accompanied by (ii) reported X-ray structure of both apo and holo formats. We compared amino acid sequences as a function of substrate affinity, including in the analysis high-affinity isozymes (low K_m_) from *Escherichia coli* [11], *Mycobacterium tuberculosis* [17], and *Arabidopsis thaliana* (thale cress; this study) [18] and low-affinity isozymes (high K_m_) from *Saccharomyces cerevisiae* (yeast) [19] and *Homo sapiens* (human) [15] (Fig. 1c; Table 1).

**Table 1:** Comparison of secondary structures and interactions at the ligand-binding site

	*A*. *thaliana*	*S. cerevisiae*	*H. sapiens*	*E. coli*	*M*. *tuberculosis*
PDB ID	4OOP	3P48	2HQU	1RN8	2PY4
Sequence similarity^a^	Self	56.2 %	63.9 %	35.5 %	39.2 %
*K*_m_	0.4 ± 0.1 μM	13.2 ± 0.6 μM	3.6 ± 1.9 μM	0.5 μM (SE 17 %)	0.46 ± 0.2 μM
α helix 1st res	S89	S69	S86	S72	S65
α helix 5th res	W93	V73	A90	H76	T69
α1st–WAT–α5th	Present	Absent	Absent	Absent	Absent
References	This study	Tchigvintsev et al. [19]	Toth et al. [15]	Barabas et al. [11]	Pecsi et al. [17]

α1st–WAT–α5th refers to the presence of an interaction among the first residue of the α helix, a water molecule, and the fifth residue of the α helix

^a^Sequence similarities between *Arabidopsis* and each species were calculated by using SIM [28] on the ExPASy server (http://www.expasy.org)

We found that *Arabidopsis* dUTPase contains a unique tryptophan at the 93rd position, which is located between motifs 2 and 3 (Fig. 1c; Table 1). To identify the role of the 93rd tryptophan, we solved the structure of *Arabidopsis* dUTPase in its holo form. This homo-trimeric enzyme shows a unique set of interactions with ligands, amino acids, and water molecules at each active site. A comparison of the active sites reveals that tryptophan 93 seems to play a key role in guiding serine reorientation via a water molecule to orient the incoming ligand. In high-affinity dUTPases, the serine residue can be held in place in similar manner by a positively charged residue located four amino acids away. In contrast, low-affinity enzymes lack charges at the corresponding sites.

## Methods

### dUTPase preparation

*Arabidopsis* dUTPase was prepared as described previously [18, 20, 21]. Briefly, His-tagged *Arabidopsis* dUTPase was purified via Ni–NTA chromatography from the cleared lysate of *E. coli* JM103 (DE3) cells. The tag was removed by thrombin cleavage. The resulting dUTPase includes three extra amino acids, Gly–Ser–His, at the amino-terminus (Fig. 1c).

### dUTPase activity assay

The enzymatic activity assay was performed using cresol red, and the K_m_ values were calculated using the integrated Michaelis–Menten method [22, 23] with a stopped-flow instrument (Hi-Tech SF-61DX2, TgK Scientific, Bradford-on-Avon, UK) equipped with a photodiode array detector. The assay solution contained 100 mM KCl, 5 mM MgCl_2_, and 0.25 mM bicine at pH 7.6. dUTPase, to a final concentration of 50 nM, was rapidly mixed with 1–5 μM dUTP solutions in the stopped-flow system, and absorbance was monitored at 573 nm (Fig. 1d).

### Crystallization

Crystals of holo *Arabidopsis* dUTPase were grown by vapor diffusion with the hanging drop method using 2 M ammonium sulfate as a precipitant in 50 mM Tris–HCl at pH 7.4 and 5 mM of the non-hydrolyzable ligand analog 2′-deoxyuridine 5′-[(α,β)-imido]-triphosphate (dUpNHpp; Jena Bioscience, Jena, Germany) along with 5 mM MgSO_4_ (Table 2) [18, 20, 21].

**Table 2:** Data collection and structural refinements of *Arabidopsis* dUTPase

Parameters	Apo dUTPase	Holo dUTPase
PDB ID	4OOQ	4OOP
System	Orthorhombic	Orthorhombic
Space group	P212121	P212121
Unit cell dimensions (Å)		
a	69.9	70.1
b	70.6	70.7
c	75.0	75.1
Data collection		
Wavelength (Å)	1.542	0.978
Resolution (scaling) range (Å)	20.34–2.00 (2.1–2.0)	31.97–1.5 (1.55–1.5)
No. of observed reflections	209,897	360,703
No. of unique reflections used	25,874	59,499 (5804)
*I/s* (*I*)	41.6 (18.5)	46.4 (8.6)
Completeness (%)	99.2 (98.5)	95.0 (90.0)
R_merge_ (%)	7.6 (17.0)	7.0 (32.0)
Refinement		
Resolution range (Å)	20.34–2.00 (2.1–2.0)	31.97–1.5 (1.55–1.5)
R_work_ (%)	14.8	17.3
R_free_ (%)	19.6	20.8
No. of non-hydrogen atoms	3287	3658
No. of water molecules	345	552
RMS deviations from ideal values		
Bond lengths (Å)	0.007	0.007
Bond angles (°)	1.08	1.16
Mean B value (Å^2^)	22.0	20.0
Interpretable residues (out of 166)		
Chain A	26–156	25–157
Chain B	25–154	25–166
Chain C	26–152	26–152

Values in parentheses correspond to the highest-resolution shell

R_merge_ = Σ*|I*_*obs*_ *−* 〈* I* 〉*|/*Σ*I*_*obs*_, where *I*_*obs*_ and 〈* I* 〉 are the observed intensity and the mean intensity of the reflection, respectively

R_work_ = Σ||F_obs_| − |F_calc_||/Σ|F_obs_|

R_free_ values are collected for a randomly selected 5 % of the data that was excluded from the refinement

### X-ray diffraction data collection, structural analysis, and structural mining

Diffraction data were collected from a single holo dUTPase crystal at the Advanced Photon Source (Argonne, IL, USA) sector 14-BM-C. The data collection and refinement statistics are shown in Table 2. While the holo crystal diffracted beyond 1.2 Å resolution, we only used reflections up to 1.5 Å resolution to keep the redundancy more than five and the linear R factor less than 0.6. To increase the resolution of the structure in apo formats, we further refined the apo structure (PDB ID, 2P9O) with previously collected data by PHENIX [24]; the updated apo structure was used as the starting model for the structure in the holo format. The structure of the holo enzyme was also refined by PHENIX using the same Rfree flag assignment used in apo structure refinement. We deposited the apo and holo structures in PDB with the IDs 4OOQ and 4OOP, respectively. The COOT [25], PISA [26], and PyMOL (Schrödinger, San Diego, CA) software packages were used for structural mining and graphical presentation.

## Results and discussion

### Enzymatic activity and crystallization

The *Arabidopsis* dUTPase showed enzymatic activity, with estimated K_m_ and V_max_ values of 0.4 ± 0.1 μM and 1.2 ± 0.05 µM s^−1^, respectively, at pH 7.6 and 25 ℃ (Fig. 1d). Therefore, the enzyme belongs to the high-substrate-affinity group, which also includes the *E. coli* and *M. tuberculosis* proteins (Table 1).

Crystallization of the holo *Arabidopsis* dUTPase was performed using the non-hydrolysable dUTP analog dUpNHpp and ammonium sulfate as the precipitant. In the apo *Arabidopsis* dUTPase, taurine was an indispensable additive for the growth of single crystals [18]. In the holo format, the growth of single crystals was not dependent on the presence of taurine. The protein formed needle clusters in the absence of dUpNHpp and taurine.

### *Arabidopsis* dUTPase structure

**Fig. 2:**
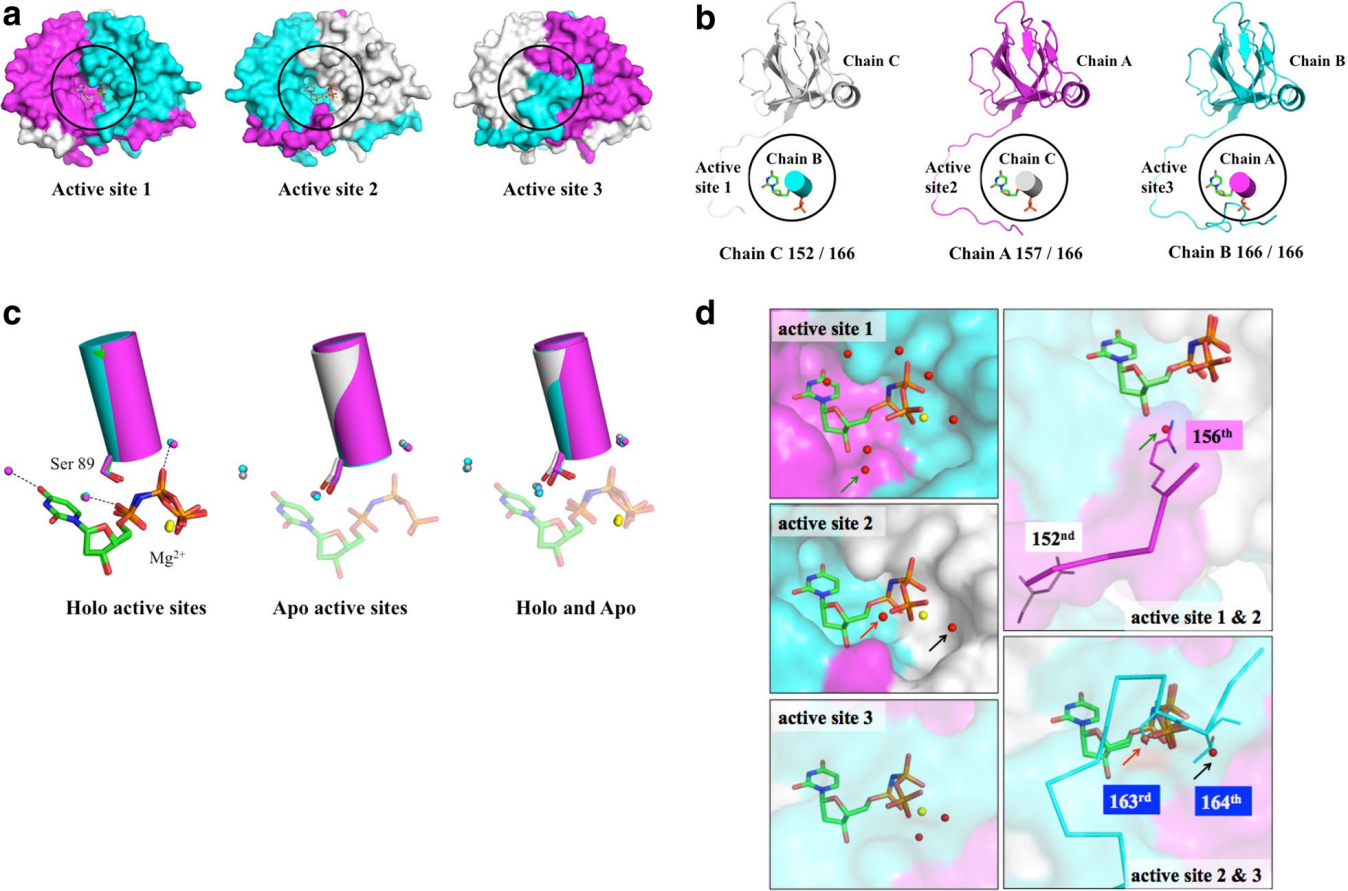
The structures of the apo and holo forms of *Arabidopsis* dUTPase. **a** Surface presentation of the dUTPase trimer. *Circles* indicate the active sites. Chains A, B, and C are colored in *magenta*, *cyan*, and *gray*, respectively. **b** The structure of each active site is composed of three subunits. Each diagram contains a ligand, an α-helix carrying Ser89 and Trp93, and a subunit that provides the C-terminal residues to the active site in a cartoon model. The lengths of the C-termini of the subunits are different. **c** Apo and holo common water molecules at the active site. Stick models represent the ligand and the ligand-interacting serine residue (S89). **d** Ligand interaction–replacement by water molecules and amino acids. The water molecules indicated by the *arrow* are initially associated with the ligand and later potentially replaced with C-terminal amino acids

The refined *Arabidopsis* dUTPase structure showed a trimeric structure (Figs. 1a, 2a; Table 2). The first 24 N-terminal residues of all of the subunits were equally disordered. However, there were notable differences in the interpretable C-terminal domains when all of the active sites had a bound ligand (Fig. 2b). Next, we used PISA to assess the effects of crystal packing on the different C-terminal lengths [26]. Chains A and C interacted with neighboring subunits (Additional file 1: Figure S1), while chain B did not. Since α carbon positions between chain B and C are identical, crystal packing apparently contributes to the variety in the chain A C-terminal length.

### Three apo–holo common waters accommodate the substrate

Because the C-terminal residues are involved in ligand interactions, the environment of the ligand-binding site was analyzed (Additional file 2: Table S1 and Additional file 3: Figure S2). Aside from the three ordered water molecules that interact with the magnesium ion, there were three additional ordered water molecules (Fig. 2c) that interact with the ligand at the nitrogenous base oxygen atom O_4_, the α-phosphate oxygen atom, and the β-phosphate oxygen atom. When the active sites of both the apo and holo forms are superimposed, all of the apo active sites have three water molecules at similar locations as those commonly found in the holo form. These data suggest that the coordination of these water molecules is necessary for initial ligand binding at the active site.

### Replacement of ligand-associated water with C-terminal residues reorients the ligand

Ligand 1 bound to active site 1 involves the shortest interpretable C-terminus (Fig. 2d, active site 1) and has the most interactions with water molecules. Ligand 2 in active site 2 involves a medium-length interpretable C-terminus, and has the fewest interactions with water molecules and amino acid residues. Ligand 2 has the highest average B-factor among the 3 ligands, namely 42.8 Å^2^ as calculated by COOT. A likely explanation for this result is that the C-terminal residues involved in this active site have the most interactions with neighboring subunits due to crystal packing, as discussed above (Additional file 1: Figure S1). Superimposition of the ligand 1 and 2 binding sites (Fig. 2d, active sites 1 and 2) shows that the ligand 1-interacting water molecule (indicated by a green arrow in Fig. 2d) is located at a coordinate similar to that of the Arg156 amino group in the ligand 2 binding site. The electron density map does not support the presence of the corresponding Arg156 coordinate in the ligand 1 binding site. We interpret these data as ligand 1 being in a pre-ordered state, such that the bound ligand is reoriented by the replacement of the ligand–water interaction with the ligand–Arg156 interaction.

This “interaction–replacement” phenomenon is also observed in the ligand 3 binding site (red and black arrows in Fig. 2d, active sites 2 and 3). This binding site involves the completed C-terminus and has the largest number of ligand-amino acid interactions. A superimposition of all of the ligand-binding sites shows that Arg156 undergoes a structural change to interact with the water molecule ordered by the magnesium ion in the holo form. Some ligand–water molecule interactions found in both the ligand 1 and 2 binding sites are replaced by ligand–Ser163 or ligand–Thr164 interactions in the ligand 3 binding site.

We compared ligand coordinates focusing between ligand 1 and 3 binding sites. It was because the C-terminal residues involved in the ligand 2 binding site (chain A residues from 145 to 152) had interactions with four neighboring residues, and resulted the structural difference in the C-termini residues comparing from other subunits, chain B and C (Additional file 1: Figure S1). It appears that the ligand coordinate in the ligand 3 binding site appears to be engaged in the more stable orientation in terms of increased ligand–amino acid interactions. Additionally, the ligand coordinate is in the most favorable position for nucleophilic attack. The γ-phosphate group of ligand 3 occupies a position that is different from those of the other two ligands. A comparison of the ligand 3 and 1 coordinates relative to the catalytic water shows that the ligand 3 α-phosphate is closer to the catalytic water by approximately 0.3 Å (W_cat_ in Fig. 1a, inhibitor-bound active site).

### Roles of Trp93 in *Arabidopsis* dUTPase ligand binding

**Fig. 3:**
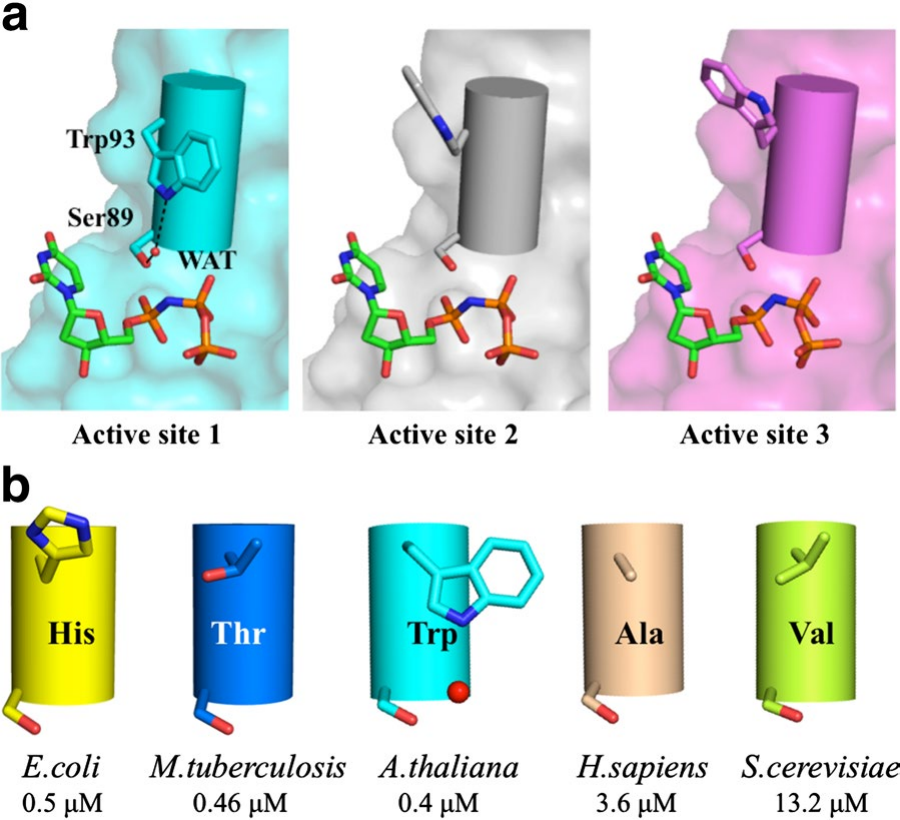
Interactions between serine 89 and tryptophan 93 in *Arabidopsis* dUTPase. **a** Location of the Trp93 side chain in each subunit at different stages of substrate orientation. **b** Comparison of amino acids in other dUTPases that correspond to Ser 89 and Trp 93 in *Arabidopsis* dUTPase

Serine 89 of motif 2 in *Arabidopsis* dUTPase is an important residue for maintaining a reaction-favorable ligand orientation at the active site (Fig. 2c). It undergoes a conformational change between the apo and holo forms to interact with the oxygen atom between α,β-phosphate [10]. This serine side chain flipping is observed in the obtained *Arabidopsis* dUTPase structure (Figs. 2c, 3a). This rearrangement is commonly observed in all active sites of the compared dUTPases except for one of the active sites in yeast dUTPase.

Tryptophan 93 of *Arabidopsis* dUTPase may play an important role in orienting serine 89 in the holo form (Fig. 2c). In the apo form, all of the tryptophan 93 side-chain coordinates were oriented upwards; thus, all of the tryptophan 93 N^ε1^ atoms were located away from the serine 89 O^γ^. These are likely due to crystal-packing induced hydrophobic interactions with proline 46 in neighboring subunits. In contrast, the tryptophan 93 side chain in the holo-form chain B had different coordinates compared with those in other two subunits. It is located such that the tryptophan 93 N^ε1^ was closer to the serine 89 O^γ^. Although tryptophan 93 in the holo form may make the same hydrophobic interactions with proline 46 in neighboring subunit, this particular orientation was likely due to the presence of a nearby water molecule, which bridges its interaction with serine 89. This serine 89–water–tryptophan 93 interaction was only found in chain B. This is part of the ligand 1 binding site, which involves the shortest interpretable C-terminus.

Together with the finding that ligand 1 is likely in a pre-ordered state for the reaction, we assume that tryptophan 93 acts as a key residue for initial ligand orientation at the active site by promoting serine 89 side-chain coordinate changes by interacting with the ordered water molecule.

### Molecular mechanism for the differences in ligand affinity

*Arabidopsis* dUTPase belongs to the high-affinity group, along with the *E. coli* and *Mycobacterium* dUTPases (Table 1; Fig. 2c). Tryptophan 93 of *Arabidopsis* dUTPase holds serine 89 in place via a water molecule and forms a favorable conformation for substrate binding. It appears that the high-affinity dUTPases from species such as *E. coli* and *Mycobacterium* have charged or polar amino-acid substitutions corresponding to tryptophan 93 in *Arabidopsis* dUTPase. In contrast, the low-affinity dUTPases from humans and yeast have non-polar amino-acid substitutions. Additionally, chain C of the yeast dUTPase has the motif 2 serine residue whose side-chain oxygen atom is located away from the nitrogen atom between α,β-phosphate, and yeast dUTPase has the highest K_m_ value among the five dUTPases compared in this study. These data suggest that the amino-acid substitution affects the hydration state at the active site and may influence ligand-binding affinity.

## Conclusions

The structure of *Arabidopsis* dUTPase has been analyzed. Interestingly, this homotrimeric enzyme shows varying binding site environments with respect to their types of ligand interactions. Additionally, the tryptophan 93 substitution seems to use ordered water molecules to aid in coordinating Ser89 for initial ligand binding.

## Additional files

10.1186/s13104-015-1760-1 Effect of crystal packing on C-terminal residue coordinates.
10.1186/s13104-015-1760-1 List of common ligand interactions.
10.1186/s13104-015-1760-1 Common ligand interactions at the active site.
