# Supplementary material for: Structural insights into the mechanism defining substrate affinity in Arabidopsis thaliana dUTPase: the role of tryptophan 93 in ligand orientation
Source: BMC Res Notes. 2015 Dec 15;8:784. doi: 10.1186/s13104-015-1760-1 (PMC4678481; doi:10.1186/s13104-015-1760-1)
Supplement: Supplementary file 1 — 10.1186/s13104-015-1760-1 Effect of crystal packing on C-terminal residue coordinates. [file 13104_2015_1760_MOESM1_ESM.docx]

**A**
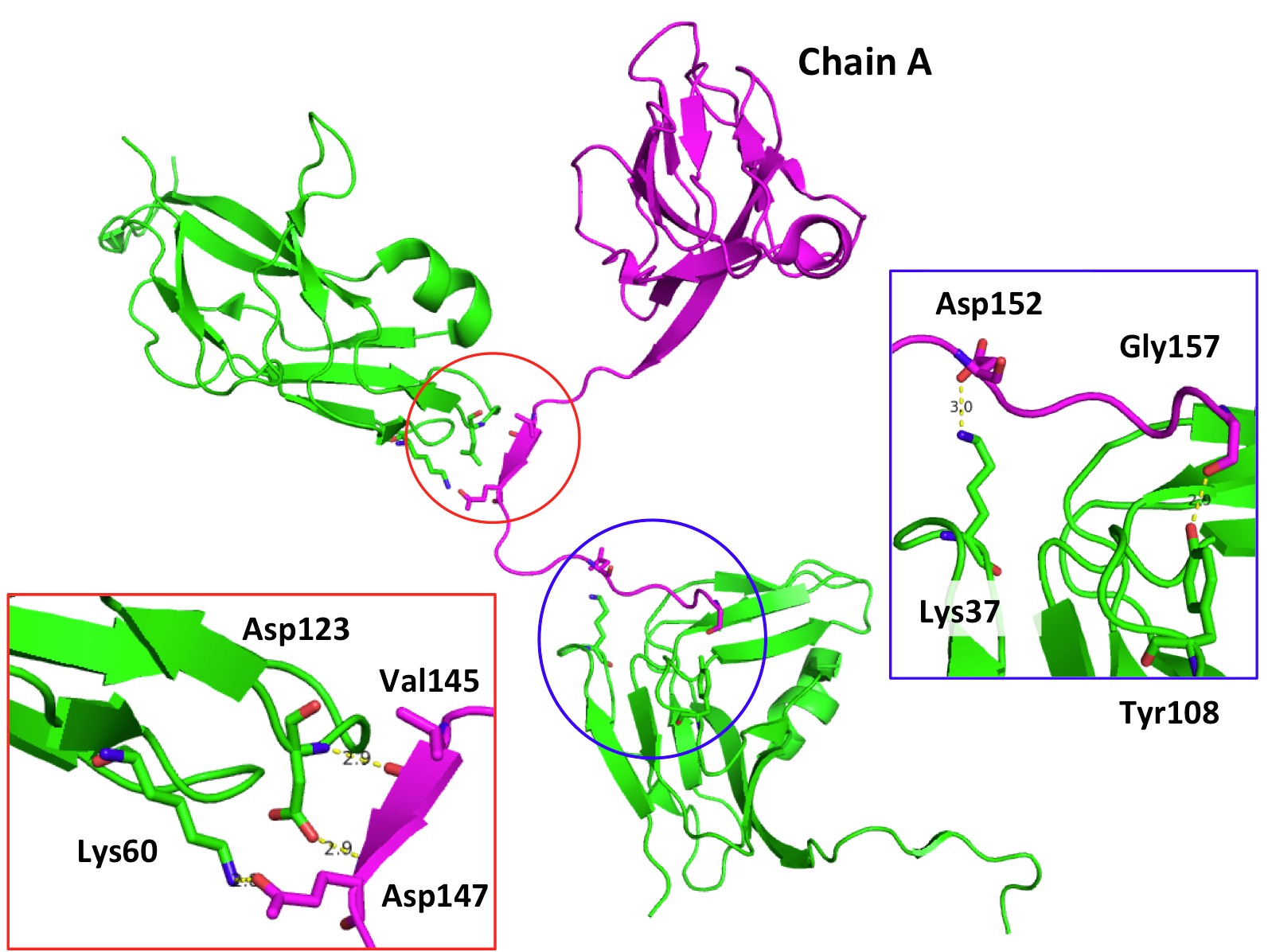
 **B**
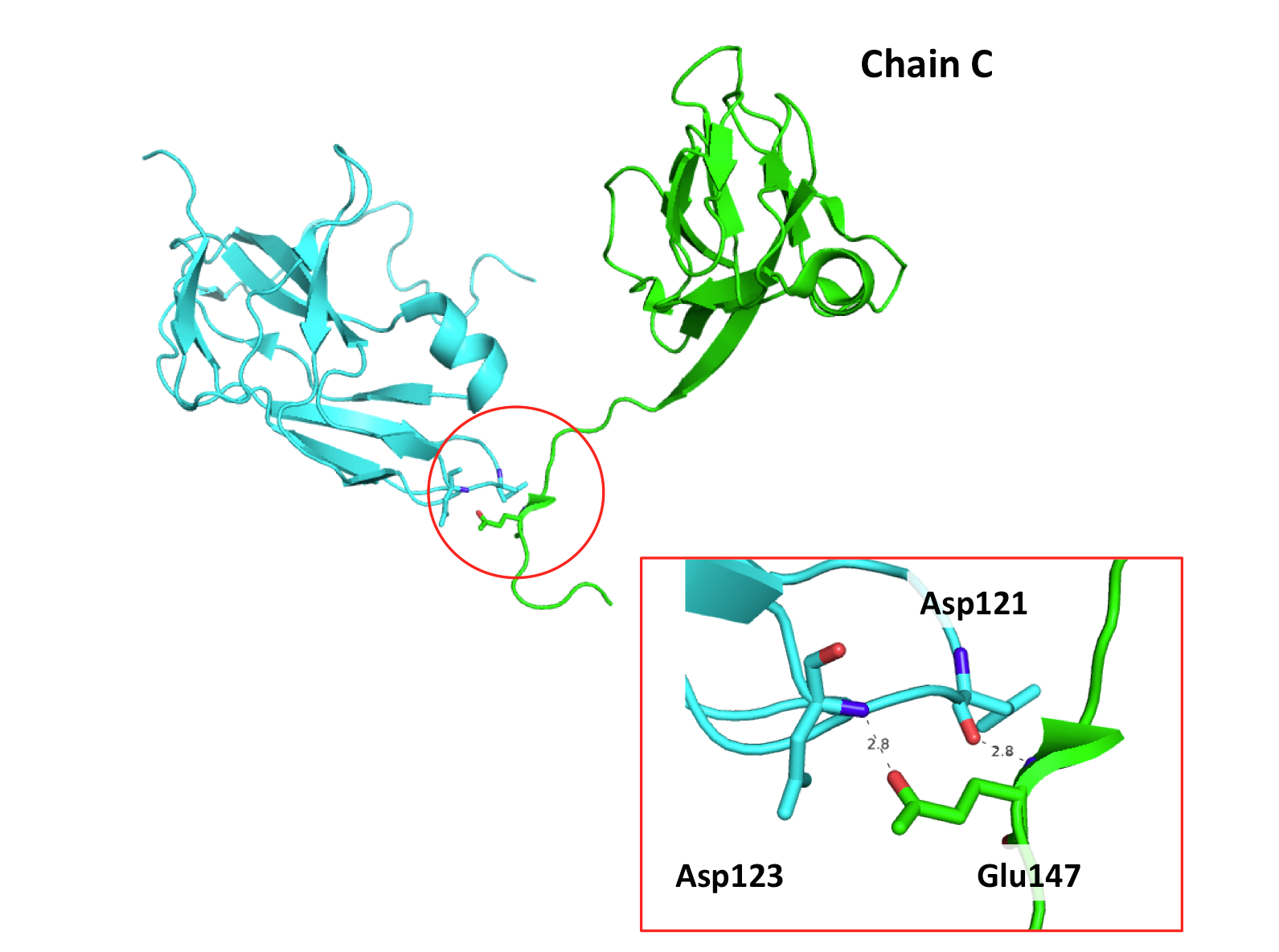


**Figure S1.** Effect of crystal packing on C-terminal residue coordinates. (A) Intersubunit interactions between chain A and neighboring subunits. Intersubunit interactions were analyzed with Proteins, Interfaces, Structures, and Assemblies (PISA) [56]. Chain A is colored magenta; its interacting subunits were generated by symmetry operations and are colored green. The locations of the interacting residues are circled, and the details of the interactions are shown in the box with the corresponding color**.** (B) Intersubunit interactions between chain C and a neighboring subunit. Chain C is colored green, and its interacting subunit is colored cyan. The location of the interaction is circled, and the details of the interaction are shown in the box.
