# Supplementary material for: Structural insights into the mechanism defining substrate affinity in Arabidopsis thaliana dUTPase: the role of tryptophan 93 in ligand orientation
Source: BMC Res Notes. 2015 Dec 15;8:784. doi: 10.1186/s13104-015-1760-1 (PMC4678481; doi:10.1186/s13104-015-1760-1)
Supplement: Supplementary file 2 — 10.1186/s13104-015-1760-1 List of common ligand interactions. [file 13104_2015_1760_MOESM2_ESM.docx]

**Table S1. Common ligand-amino acid and ligand-water molecule interactions**

| Ligand 1  (A201) | Interacting  atoms | Distance (Å) | | Ligand 2  (B202) | Interacting  atoms | Distance (Å) | | Ligand 3  (B203) | Interacting  atoms | Distance (Å) |
| --- | --- | --- | --- | --- | --- | --- | --- | --- | --- | --- |
| A201_O2 | A113_GLY_N | 3.08 |  | B202_O2 | B113_GLY_N | 3.12 |  | B203_O2 | C113_GLY_N | 3.18 |
| A201_O4 | A102_GLY_N | 2.85 |  | B202_O4 | B102_GLY_N | 2.90 |  | B203_O4 | C102_GLY_N | 2.84 |
| A201_O4 | A308_HOH_O | 2.79 |  | B202_O4 | B301_HOH_O | 2.80 |  | B203_O4 | B303_HOH_O | 2.79 |
| A201_N3 | A113_GLY_O | 2.80 |  | B202_N3 | B113_GLY_O | 2.78 |  | B203_N3 | C113_GLY_O | 2.81 |
| A201_O3' | A105_ASP_OD2 | 2.83 |  | B202_O3' | B105_ASP_OD2 | 2.66 |  | B203_O3' | C105_ASP_OD2 | 2.65 |
| A201_O3' | A105_ASP_N | 3.05 |  | B202_O3' | B105_ASP_N | 3.19 |  | B203_O3' | C105_ASP_N | 3.10 |
| A201_O1A | B134_GLN_NE2 | 2.97 |  | B202_O1A | C134_GLN_NE2 | 2.88 |  | B203_O2A | A134_GLN_NE2 | 2.65 |
| A201_O1A | A303_HOH_O | 2.73 |  | B202_O1A | B505_HOH_O | 2.82 |  | B203_O2A | B307_HOH_O | 2.64 |
| A201_O2A | B134_GLN_NE2 | 3.00 |  | B202_O2A | C134_GLN_NE2 | 2.90 |  | B203_O2A | A134_GLN_NE2 | 2.60 |
| A201_O2A | A449_HOH_O | 2.90 |  | B202_O2A | B382_HOH_O | 2.71 |  | B203_O1A | B349_HOH_O | 2.77 |
| A201_O2A | B463_HOH_O | 2.86 |  | B202_O2A | B408_HOH_O | 2.82 |  | B203_O1A | A323_HOH_O | 2.86 |
| A201_O2A | A447_HOH_O | 2.80 |  | B202_O2A | B464_HOH_O | 2.76 |  | B203_O1A | B405_HOH_O | 2.78 |
| A201_N3A | B89_SER_OG | 2.57 |  | B202_N3A | C89_SER_OG | 2.41 |  | B203_N3A | A89_SER_OG | 2.75 |
| A201_O1B | B90_GLY_N | 2.89 |  | B202_O1B | C90_GLY_N | 2.94 |  | B203_O2B | A90_GLY_N | 2.87 |
| A201_O1B | A318_HOH_O | 2.68 |  | B202_O1B | B325_HOH_O | 2.83 |  | B203_O2B | B320_HOH_O | 2.60 |
| A201_O2B | B88_ARG_NE | 2.83 |  | B202_O2B | C88_ARG_NE | 2.85 |  | B203_O1B | A88_ARG_NE | 2.77 |
| A201_O2B | B88_ARG_NH2 | 3.06 |  | B202_O2B | C88_ARG_NH2 | 2.98 |  | B203_O1B | A88_ARG_NH2 | 3.02 |
| A201_O2B | B366_HOH_O | 3.00 |  | B202_O2B | C339_HOH_O | 3.05 |  | B203_O1B | A393_HOH_O | 3.07 |
| A201_O2B | B463_HOH_O | 2.91 |  | B202_O2B | B408_HOH_O | 2.95 |  | B203_O1B | A323_HOH_O | 2.94 |
| A201_O1G | B366_HOH_O | 2.87 |  | B202_O1G | C339_HOH_O | 2.91 |  | B203_O1G | A393_HOH_O | 2.87 |
| A201_O1G | A447_HOH_O | 2.86 |  | B202_O1G | B464_HOH_O | 3.01 |  | B203_O1G | B405_HOH_O | 3.07 |

**Table B. Common water-amino acid interactions at active site**

| Ligand 1  water | Interacting  atoms | Distance (Å) | | Ligand 2  water | Interacting  Atoms | Distance (Å) | | Ligand 3  water | Interacting Atoms | Distance (Å) |
| --- | --- | --- | --- | --- | --- | --- | --- | --- | --- | --- |
| A308_HOH_O | A100_GLY_O | 2.83 |  | B301_HOH_O | B100_GLY_O | 2.86 |  | B303_HOH_O | C100_GLY_O | 2.88 |
| A303_HOH_O | B87_PRO_O | 2.86 |  | B505_HOH_O | C87_PRO_O | 2.88 |  | B307_HOH_O | A87_PRO_O | 2.91 |
| A303_HOH_O | A103_VAL_N | 3.04 |  | B505_HOH_O | B103_VAL_N | 3.03 |  | B307_HOH_O | C103_VAL_N | 2.98 |
| A449_HOH_O | A105_ASP_OD2 | 2.86 |  | B382_HOH_O | B105_ASP_OD2 | 2.95 |  | B349_HOH_O | C105_ASP_OD2 | 2.85 |
| A449_HOH_O | A105_ASP_OD1 | 2.97 |  | B382_HOH_O | B105_ASP_OD1 | 3.30 |  | B349_HOH_O | C105_ASP_OD1 | 2.94 |
| B341_HOH_O | A105_ASP_OD1 | 2.86 |  | B343_HOH_O | B105_ASP_OD1 | 2.60 |  | C337_HOH_O | C105_ASP_OD1 | 2.60 |
| A308_HOH_O | A115_ILE_N | 2.86 |  | B301_HOH_O | B115_ILE_N | 2.84 |  | B303_HOH_O | C115_ILE_N | 2.84 |
| B463_HOH_O | B134_GLN_OE1 | 2.90 |  | B408_HOH_O | C134_GLN_OE1 | 2.90 |  | A323_HOH_O | A134_GLN_OE1 | 2.86 |
| B366_HOH_O | B52_ASP_OD2 | 2.60 |  | C339_HOH_O | C52_ASP_OD2 | 2.71 |  | A393_HOH_O | A52_ASP_OD2 | 2.74 |
| B463_HOH_O | B52_ASP_OD1 | 2.65 |  | B408_HOH_O | C52_ASP_OD1 | 2.84 |  | A323_HOH_O | A52_ASP_OD1 | 2.68 |
| B463_HOH_O | B134_GLN_OE1 | 2.90 |  | B408_HOH_O | C134_GLN_OE1 | 3.14 |  | A323_HOH_O | A134_GLN_OE1 | 2.90 |
| A318_HOH_O | B91_LEU_N | 2.90 |  | B325_HOH_O | C91_LEU_N | 3.00 |  | B320_HOH_O | A91_LEU_N | 2.90 |
| A449_HOH_O | B134_GLN_OE1 | 3.20 |  | B382_HOH_O | C134_GLN_OE1 | 3.20 |  | B349_HOH_O | A134_GLN_OE1 | 3.02 |
| A449_HOH_O | A103_VAL_O | 3.16 |  | B382_HOH_O | B103_VAL_O | 3.20 |  | B349_HOH_O | C103_VAL_O | 3.13 |

Ligands are referred by their chain ID and residue numbers used in the PDB file. Atoms interacting with ligands are indicated by the following description; chain ID, residue number, residue name, and interacting atom name. The distances were obtained by COOT. The underlined water molecules are ones directly interacting with magnesium ions at the active sites. Gray highlighted water molecules are catalytic water molecules.
