# Supplementary material for: Structural insights into the mechanism defining substrate affinity in Arabidopsis thaliana dUTPase: the role of tryptophan 93 in ligand orientation
Source: BMC Res Notes. 2015 Dec 15;8:784. doi: 10.1186/s13104-015-1760-1 (PMC4678481; doi:10.1186/s13104-015-1760-1)
Supplement: Supplementary file 3 — 10.1186/s13104-015-1760-1 Common ligand interactions at the active site. [file 13104_2015_1760_MOESM3_ESM.docx]

**
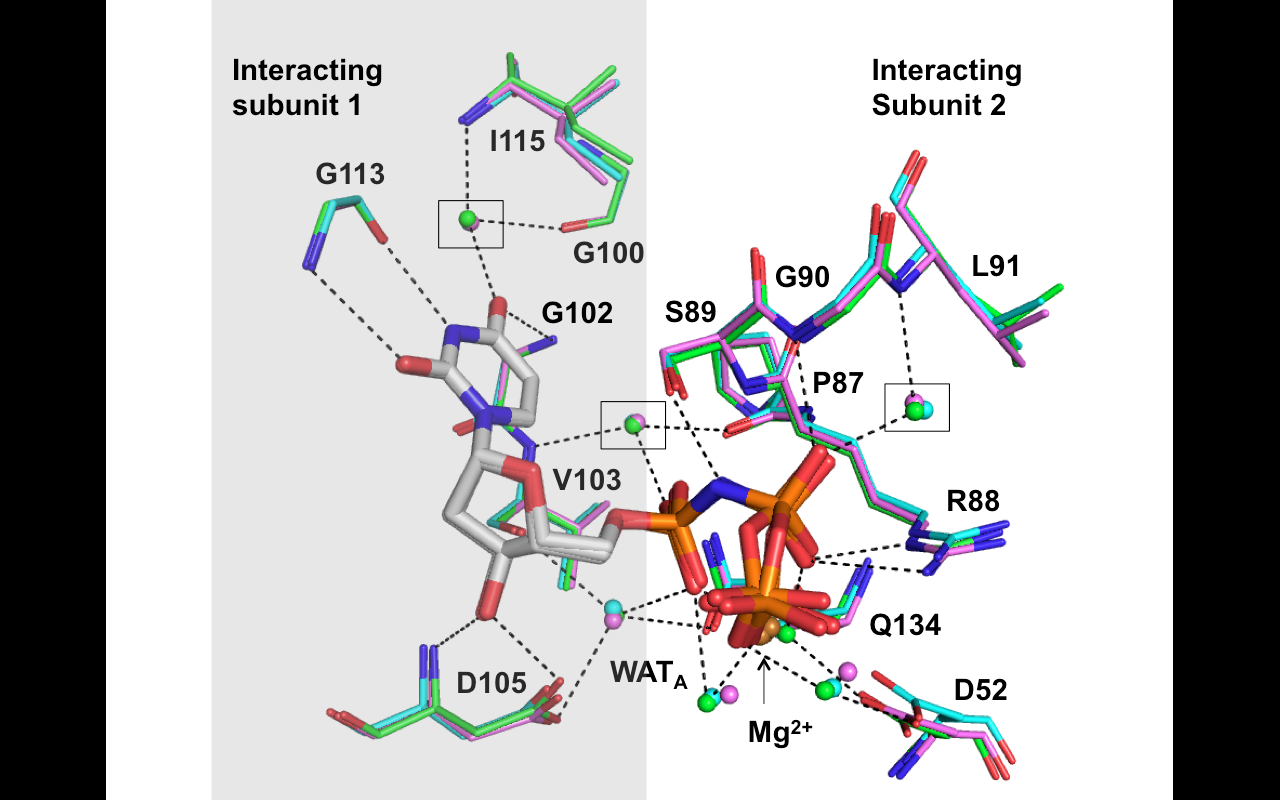
**

**Figure S2.** Common ligand interactions at the active site. Superimposed diagram showing common ligand interactions. All 3 ligands, the catalytic water molecules (WAT_A_), the ligand-interacting water molecules, and the amino acid residues were aligned using the N1, N3, and C5 atom coordinates from the nitrogenous base of the ligand. The carbons in chains A, B, and C are colored magenta, cyan, and green, respectively. Spheres represent the interacting waters at each active site, and the same colored spheres belong to each binding site as indicated in Figure 2B (magenta for the Ligand 1 binding site, cyan for the Ligand 2 binding site, and green for the Ligand 3 binding site). Because common ligand interactions involve 2 neighboring subunits, the residues in the gray shaded area belong to a single subunit (interacting subunit 1). The residues in the white area belong to the other subunit (interacting subunit 2). The black dotted lines indicate polar contacts (details in Table S1). Among the water molecules that were common to both the apo and holo forms, those discussed in the main text are indicated by black boxes.
